# Notable correlation between serum epidermal growth factor values and inflammatory status in patients with COVID‐19

**DOI:** 10.1002/iid3.1355

**Published:** 2024-08-07

**Authors:** Héctor José Pérez, Tania Crombet

**Affiliations:** ^1^ Critical Care Division Saturnino Lora Provincial Hospital Santiago de Cuba Cuba; ^2^ Clinical Trials Division Centre for Molecular Immunology Havana Cuba

**Keywords:** COVID‐19 biomarkers, epidermal growth factor, serum biomarkers

## Abstract

**Introduction:**

Despite its crucial role in Epidermal Growth Factor Receptor (EGFR) activation, and the resulting impact on the health‐disease process, epidermal growth factor (EGF) is an underexplored molecule in relation to how its serum concentrations relate to other analytes and clinical variables in pathological contexts.

**Objective:**

To clarify the possible correlation between EGF and clinical and analytical variables in the context of COVID‐19.

**Methods:**

Cross‐sectional observational and analytical study, in patients with virological and clinical diagnosis of COVID‐19, selected by simple random sampling, admitted between August and September 2021. UMELISA‐EGF commercial kits were used.

**Results:**

Differences in overall EGF values were observed between groups (566.04 vs. 910.53 pg/ml, *p* = .0430). In COVID‐19 patients, no notable correlations were observed for neutrophil, platelet, triglyceride or liver enzyme values (*p* > .05). Significant correlations were observed with the neutrophil‐lymphocyte indicator (r = 0.4711, *p* = .0128) as well as with the platelet‐lymphocyte index (r = 0.4553, *p* = .0155). Statistical results of multivariate regression analysis suggest NLR (*β* = .2232, *p* = .0353) and PLR (*β* = .2117, *p* = .0411) are predictors of inflammation in patients with COVID‐19.

**Conclusions:**

Serum EGF concentrations in COVID‐19 correlate positively with prognostic inflammatory markers of severity and could presumably act as an independent risk factor for the development of inflammation in response to new severe acute respiratory syndrome coronavirus 2 (SARS‐CoV‐2).

## Graphical abstract

We pioneered the potential implications of epidermal growth factor in the context of lung inflammation, as well as potential correlates with other widely used studies in clinical medicine, and the ability of this molecule to behave as a predictor of severity in the context of COVID‐19, with a view to obtaining real‐world data that will help understand the implications of this molecule in the development of the lung environment. health‐disease process, based on serum determinations of this protein, which would allow a low‐cost generalization for health systems.

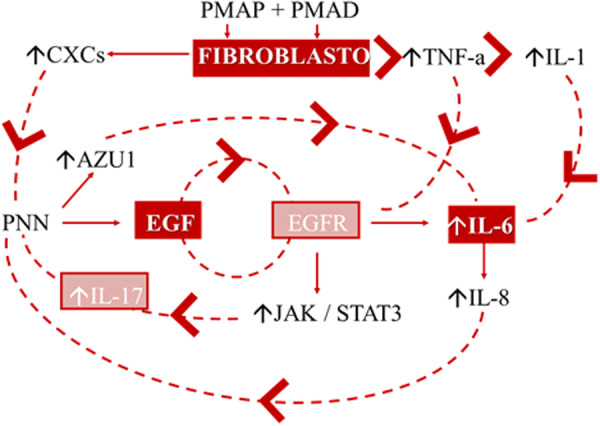

## 1 INTRODUCTION

The health crisis triggered by the new severe acute respiratory syndrome coronavirus 2 (SARS‐CoV‐2) has been one of the greatest challenges to global health. Together with the emergence of variants and the ineffective policies that led to global vaccination,^1^ the risks of re‐emergence and subsequent emergence of new pandemic outbreaks are still a latent risk.

Although the most mediated means of access of SARS‐CoV‐2 to cells is the Angiotensin‐coverting enzyme 2 (ACE2) protein,^1^ other promising candidates have been identified as possible points of internalization, receptors overexpressed in the inflammatory environment with a direct link to the presence of tumoral necrosis factor (TNF), interleukin (IL)1 and IL‐6,^2^, ^3^ the latter being strongly associated with the development of severe/critical forms of the disease; which is in turn stimulated by EGFR activation.^4^

EGFR has as its main ligand a small molecule, of only 53 amino acids, called epidermal growth factor (EGF).^5^, ^6^ This molecule could not only be an important mediator in COVID‐19 because of its direct relationship with Epidermal Growth Factor Receptor (EGFR). Transcriptomic analyses point to an interesting correlate between EGF and IL‐6, in the context of neutrophil activity,^7^ a crucial player in the anti‐SARS‐CoV‐2 immune response.

Previous studies had already established a correlation between EGF and IL‐8,^8^ a critical cytokine in neutrophil recruitment. More recently in the context of COVID‐19 they have reported similar trends, extending the observations to other classical pro‐inflammatory cytokines such as IL‐1β and macrophage inflammatory protein (MIP) 1a (CCL3), as well as cytokines associated with Th2 (IL‐4, IL‐5, and IL‐13) and Th17 (IL‐17A, IL‐17F, IL‐17E/IL‐25, and IL‐22) polarization.^9^ All of which points to an effect of EGF beyond the direct interaction with its classical receptor.

EGF‐dependent signaling has been associated with markedly inflammatory conditions.^10^ The role it can play is highly complex, although its negative regulatory role of cytokines such as TNF and INF.g has been reported in specific tissues;^11^, ^12^ The large body of evidence indicates a role more closely related to the activation of inflammatory response patterns.^13^, ^14^ Covid‐19 generally begins with flu‐like symptoms and can be asymptomatic or have a mild to severe course.^15^ The basis of its pathophysiology is characterized by a significant load of inflammation; Studies have revealed the complex network of related molecular and cellular signals, and it has been reported that the NLR and the proportion of platelets and lymphocytes are related to the infection and its progress.^16^, ^17^, ^18^, ^19^, ^20^ Although a growing number of studies suggest that EGF is involved in multiple inflammatory processes as part of the immune response to pathogens, field data on the serum behavior of the protein in humans in these contexts and its possible correlates are scarce.

The purpose of our current research is (1) to explore the relationship between EGF and severe COVID‐19 and (2) to clarify whether evidence suggests that EGF could be a biomarker of COVID‐19 severity, aspects that have not been reported, based on serum studies, in previous studies. Clarifying the possible correlation between EGF and COVID‐19 will provide new possibilities for a better understanding of the inflammatory phenomenon in the context of pathogen‐triggered immune response.

## 2 PATIENTS AND METHODS

### 2.1 Study population

21 patients with severe COVID‐19, treated at the Julio Trigo Hospital and admitted between August‐September 2021, and 21 apparently healthy blood bank subjects were enrolled in the study. The diagnostic criteria used were those declared by MINSAP based on WHO recommendations. The diagnoses were made by experienced, certified professionals. In the case of healthy donors, regular analyses and an excautionary physical examination certified their status as apparently healthy subjects. This study was approved by the Research Ethics Committees of the participating institutions. This research adheres to the letter and spirit of the Declaration of Helsinki.

### 2.2 Clinical and analytical data

Clinical and analytical data were collected by professional teams during patient care or regular evaluation of the blood donor at the corresponding medical institutions.

For the measurement of biological parameters, sample collections were carried out simultaneously. The processing of serum samples for EGF measurement was carried out in a deferred manner in the certified Ultra Micro Analytical System technology laboratories of the Cuban Immunoassay Center (SUMA). The samples of the control group come from a panel of healthy subjects developed before the spread of SARS‐CoV‐2 in Cuba, under the same methodologies used in the case of COVID‐19 patients.

The information used was collected from medical records and included: age, neutrophils, lymphocytes, platelets, neutrophil‐lymphocyte ratio (NLR), platelet‐lymphocyte ratio (PLR), triacylglycerides (TAG), creatinine, lactate dehydrogenase (LDH), alanine aminotransferase (ALAT), aspartate aminotransferase (ASAT). It was verified that the acquisition of the analytical parameters was performed in strict compliance with the standardized procedures of the clinical laboratory of the Institutions and in accordance with the current regulations of the CECMED.

### 2.3 Serum EGF levels

Serum EGF detection was performed using the commercial kit UMELISA‐EGF from the Cuban Immunoassay Centre. In all cases, 5 ml of blood was collected by puncture of the cephalic vein in the flexure of the arm using disposable syringes of 10 ml capacity, with 21 G hypodermic needles, deposited in a dry test tube, obtaining the serum by the coagulum retraction technique for 4 h and centrifugation (according to the manufacturer's recommendations at 3500 rpm for 10 min at 240°C). The serum obtained was dispensed by eppendorf micropipettes into 1.5 ml eppendorf vials, after which they were stored frozen at −200°C, until processing at the SUMA laboratory, certified by CECMED, at the Molecular Immunology Centre.

### 2.4 Statistical analysis

A digital database was used to record the data, created using the technical facilities of the Excel software of the Microsoft office 2010 platform (Microsoft, USA), on a Hewlett‐Packard laptop computer. Data processing was carried out on the same technological platform. In the statistical analysis, measures of central tendency (arithmetic mean), dispersion (standard deviation and confidence interval) and Pearson's correlation coefficient were used as summary parameters. Q‐Q tests for normality and the Jarque‐Bera statistic (data not shown) were performed; in addition, the statistical significance of observable differences between groups was explored with the chi‐square test (categorical variables) or Welch's *t*‐test (continuous variables), effect size was estimated using Hedge's g‐formula. A multiple regression model was established to detect independent risk factors associated with inflammation in the context of sever COVID‐19. A two‐tailed *p*‐value of 0.05 was considered statistically significant.

### 2.5 Bias control

Inclusion of subjects, outside any possible interference with the criteria of the treating physicians, was ensured by masking by transient patient codes and by performing patient selection based on simple random sampling. To mitigate the possible influence of confirmation bias and/or selective thinking, activities were carried out to unify criteria and delimit the responsibilities of specialists in intensive care services with respect to classifying patients based on the guidelines of the Ministry of Public Health on the recommendations issued by the World Health Organization. Biases in the means of observation were mitigated by guaranteeing that the studies were carried out in laboratories certified by CECMED, and by professionals with more than 5 years of experience, and with technological equipment of the same commercial brands. Statistical processing was carried out on the basis of the constitution of a single database, previously filtered and purified. It was foreseen that extreme quantitative outliers were removed from the analysis as long as they did not represent more than 30% of the total of the analysis subgroup, no extreme quantitative outliers were observed, so no data were removed from this analysis. The operations were triplicated, confirming the reproducibility of the results. In this study, 100% of the data related to the study variables were recorded and stored, with no missing data regarding the patients who were included.

### 2.6 Ethical statement

The study was designed and conducted according to the general principles established in the documents adopted by the international community in relation to biomedical research on human subjects, contained in the Declaration of Helsinki (update of the World Medical Assembly held in Brazil, 2013), with the state regulations in force according to the requirements of the national regulatory authority (Regulation 165/2000 of the CECMED), as well as in the Good Clinical Practice Guidelines of the International Conference on Harmonization (ICH E6). The research was approved by the Research Ethics Committee of the Saturnino Lora Hospital, and the corresponding certification by the Regional Ethics Committee of the southeastern region of Cuba. Before the inclusion of each subject in the study, Informed Consent was requested and obtained. No third party funding was received for the conduct of this research, nor did the subjects involved in the study receive any payment. No conflicts of interest are declared.

## 3 RESULTS

Baseline data were collected from 21 patients with COVID‐19 and 21 apparently healthy (control) subjects, and the results are shown in Table 1. The mean age in the control group and COVID group were (52.90 ± 2.07) and (63.80 ± 5.83) years, respectively, and significant differences were observed between the groups (*p* < .05). The EGF values of the control group and COVID group were (566.04 ± 109.63) and (910.53 ± 297.21) pg/ml, respectively, and significant differences were observed between groups (*p* < .05). While the effect size between ages was large (g = 0.6995) it was not equal in magnitude between EGF values (g = 0.4563).

**Table 1:** Baseline characteristics of patients.

	COVID (*n* = 21)
Age (years)	63.80 ± 5.83
Male (*n*)	10
Neutrophil (10^9^/L)	7.77 ± 1.21
Lymphocyte (10^9^/L)	1.77 ± 0.28
Platelet (10^9^/L)	272.23 ± 19.06
NLR	5.93 ± 2.64
PLR	192.47 ± 64.65
TAG (mmol/l)	1.40 ± 0.30
Creatinine (mmol/l)	78.71 ± 19.20
LDH (mmol/l)	398.36 ± 82.18
ALAT (U/L)	54.04 ± 13.99
ASAT (U/L)	47.33 ± 13.92
EGF (pg/ml)	910.53 ± 297.21

Abbreviations: NLR, Neutrophil‐lymphocyte ratio; PLR, Platelet‐lymphocyte ratio; TAG, Triacyl glycerides; LDH, lactate dehydrogenase; ASAT, aminoaspartate transferase; ALAT, alanine aspartate transferase; EGF, epidermal growth factor; *ρ*, *ρ*‐value.

The limits observed in the continuous variables studied were: Age [37–90] years, Neutrophil [2.8–13.65] cells per 109/L, Lymphocyte [0.45–3. 11] cells per 109/L, Platelet [200–360] cells per 109/L, NLR [1.14–11.5], PLR [81.9–262.71], TAG [0.4–3.1]mmol/L, Creatinine [18–228]mmol/L, LDH [97–805]mmol/L, ALAT [16–138]U/L, and ASAT [12–129]U/L.

### 3.1 Pearson's correlation analysis

To explore the factors related to severe COVID‐19, we performed Pearson correlation analysis of EGF values and baseline data. The results of the Pearson correlation analysis are shown in Table 2. From the results of the statistical analysis, we can see that the EGF values of patients with COVID‐19 correlate significantly with the neutrophil‐lymphocyte indicator (r = 0.4711, *p* = .0128) as well as with the platelet‐lymphocyte index (r = 0.4553, *p* = .0155). The lack of correlation observed with platelet values was striking (*p* = −.0085). However, there is no obvious correlation between EGF values and neutrophils, platelets, triglycerides, liver enzymes (*p* > .05).

**Table 2:** Correlation between epidermal growth factor (EGF) and analytical parameter in COVID.

	*r*	*ρ*
Age (years)	−0.3749	0.1030
Neutrophil (10^9^/L)	0.2229	0.1533
Lymphocyte (10^9^/L)	−0.3460	0.1173
Platelet (10^9^/L)	−0.0085	0.4857
NLR	0.4711	0.0128
PLR	0.4553	0.0155
TAG (mmol/l)	0.2505	0.1237
Creatinine (mmol/l)	−0.2211	0.2060
LDH (mmol/l)	0.3916	0.0323
ALAT (U/L)	−0.0719	0.3854
ASAT (U/L)	0.0459	0.2050

Abbreviations: NLR, Neutrophil‐ lymphocyte ratio; PLR, Platelet‐lymphocyte ratio; TAG, Triacyl glycerides; LDH, lactate dehydrogenase; ASAT, aminoaspartate transferase; ALAT, alanine aspartate transferase; *ρ*, *ρ*‐value; (r) Pearson correlation coefficient.

**Graph 1:**
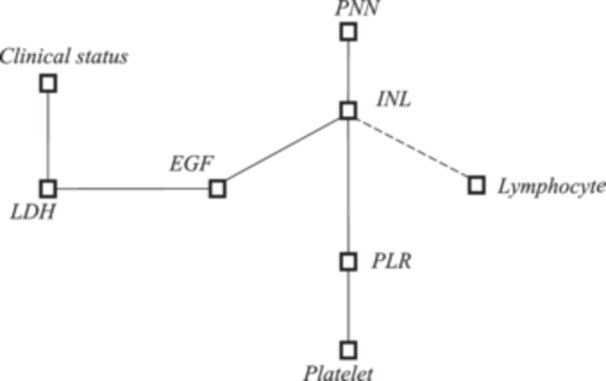
Iconography of notable correlations in relation to EGF. Legend: Neutrophil (PNN), Neutrophil‐ lymphocyte ratio (NLR), Platelet‐lymphocyte ratio (PLR), lactate dehydrogenase (LDH), epidermal growth factor (EGF), *ρ*‐value (*ρ*).

The graph of notable correlates, of interest because of their relationship with EGF, is shown in graph 1. The first order partial correlation coefficients are shown in Table 3. In the analysis of remarkable correlations, exploiting the first order partial correlation coefficients, a remarkable correlation of LDH with respect to EGF was observed, as well as of NLR with respect to EGF, and not holding the partial correlation EGF‐PLR; or Lymphocytes and Neutrophils with respect to EGF at constant NLR.

**Table 3:** First order partial correlation between epidermal growth factor (EGF) and analytical parameter in COVID.

	*rAB.C*	*ρ*
r_EGF‐PLR.NLR_	−0.0077	0.48
r_EGF‐Linf.NLR_	−0.0234	0.46
r_NLR‐PLR.EGF_	−0.6387	0.03
r_EGF‐NLR.Linf_	0.3458	0.05
r_Linf‐PLR.NLR_	−0.2313	0.19
r_Plaq‐NLR.PLR_	−0.2151	0.21
r_Plaq‐PLR.NLR_	0.3640	0.04
r_PNN‐Linf.NLR_	−0.0193	0.46
r_PNN‐NLR.Linf_	0.6165	0.002

*Note*: (rAB.C) Partial first‐order correlation coefficient; (*ρ*) *ρ*‐value.

### 3.2 Multivariate linear regression

The statistical results of the multivariate regression analysis are shown in Table 4. The results of the regression analysis showed that after adjusting EGF for age, neutrophils, lymphocytes, platelets and other factors, the NLR (*β* = .2232, *p* = .0353) and PLR (*β* = .2117, *p* = .0411) values predictive of inflammation in patients with severe COVID‐19 show that serum EGF concentrations are significant predictors of inflammation in this condition.

**Table 4:** Multivariable analyses of epidermal growth factor (EGF) and analytical parameter in COVID.

	*β*	*ρ*	*95% CI*
Age (years)	0.1916	0.0535	−0.0171 to 0.0001
Neutrophil (10^9^/L)	0.0457	0.3650	−0.0011 to 0.0028
Lymphocyte (10^9^/L)	0.1435	0.0994	−0.0008 to 0.00007
Platelet (10^9^/L)	0.0006	0.9145	−0.0338 to 0.0305
NLR	0.2232	0.0353	0.0003 to 0.0082
PLR	0.2117	0.0411	0.0045 to 0.1992
TAG (mmol/l)	0.0238	0.5157	−0.0003 to 0.0006
Creatinine (mmol/l)	0.0613	0.2923	−0.0475 to 0.0151
LDH (mmol/l)	0.1336	0.1129	−0.0242 to 0.2099
ALAT (U/L)	0.0048	0.7716	−0.0269 to 0.0203
ASAT (U/L)	0.0026	0.8286	−0.0211 to 0.0260

Abbreviations: NLR, Neutrophil‐ lymphocyte ratio; PLR, Platelet‐lymphocyte ratio; TAG, Triacyl glycerides; LDH, lactate dehydrogenase; ASAT, aminoaspartate transferase; ALAT, alanine aspartate transferase; *ρ*‐value (*ρ*), (β) Regression coefficient; (95% CI) 95% confidence interval.

## 4 DISCUSSION

Although EGF has been mostly associated with cancer, in studies mostly focused on EGFR activation,^21^, ^22^ this molecule has other potential functions. Its involvement in the central nervous system is documented from periods close to the discovery and characterization of the molecule, indicating evidence that its levels are the result of local synthesis by intrinsic and blood‐derived macrophages, glial cells and neurons, and uptake from peripheral blood through circumventricular organs and probably also across the blood‐brain barrier. Its neuromodulatory action on the neuroendocrine system and its effect on suppressing food intake and gastric acid secretion have been documented.^23^ The former effect is possibly related to its structural similarity to AgPR, with which it shares most of its bridge‐forming cysteine residues. It has also been observed that EGF administration exerts a protective effect in response to spinal cord injury in different regions of the central nervous system, by regulating apoptotic and oxidative pathways.^24^

Results in the clinical setting, where wounds were treated with recombinant EGF and platelet‐rich plasma (PRP); analyzing the susceptibility profiles of S. aureus and P. aeruginosa isolates, showing that patients treated with EGF had no infections during the follow‐up period, compared to 28/43 infections in patients treated with PRP;^25^ offering an interesting perspective of an immunomodulatory effect of local EGF.

Traditionally there is a strong association with EGF production by platelets.^26^ Platelets are key components of the inflammatory system expressing hundreds of mediators, cytokines and growth factors after activation to modulate innate and adaptive immune systems.^27^, ^28^, ^29^ EGF, although a prominent member of the biologically active proteins released from stimulated platelets, is one of the least representative, when compared in volume to platelet‐derived growth factor (PDGF) AA, BB and AB, transforming growth factor beta (TGF‐β) and insulin‐like growth factor (IGF‐1), with release accelerated by higher than physiological temperatures. Likewise these most representative growth factors are higher in whole blood compared to platelet apheresis.^30^

Despite this, many authors argue that platelets are the only source of EGF in the blood, based on the studies of Oka et al. who in 1983 described that hEGF/UG appears to be released from platelets during coagulation, inferring that platelet‐associated EGF/UG may be involved in normal vascular and tissue repair and in the pathogenesis of atherosclerotic lesions.^31^ Subsequent studies replicated the initial findings in serum, where EGF appeared and increased during spontaneous blood coagulation, reaching a plateau,^32^ as described in more detail by Cemalettin Aybay et al., that the release of EGF from platelets involved an authentic 6 kDa form of EGF (40% of total serum EGF) and remaining immunoreactive fractions corresponded to 160 kDa of proEGF.^33^ The fact that EGF concentrations increased as a function of platelet rupture supports this thesis.^34^

Explaining the phenomenon, the contributions of Rui Chen et al. were essential in understanding how the release of the soluble protease ADAMDEC1 by platelets was responsible for protocoling high molecular weight (HMW) EGF resulting in the release of a single 6 kDa domain from pro‐EGF.^35^ However, our results did not show any relationship between platelets and EGF levels (r = −0.0085, *p* = .4857). In the authors’ opinion, while platelets may be a source of EGF, the available evidence alone is not sufficient to presuppose that it is the most relevant source, and contributions from tissues, especially mucosal, epithelial and endothelial, may be more illustrative.

The results of the study showed that the serum EGF level of the COVID patients was higher than that of the control population, as well as significant differences with respect to age. To clarify the possible effect of age difference on EGF values, the control in this study was compared with results published in the literature, exploring the effect size of possible differences. Mathilde et al, for healthy women over 50 years of age (*n* = 221) reported a mean of 674 pg/ml with a standard deviation of 289 pg/ml,^25^ no differences were observed with respect to our group of women (g = 0.0071). In the case of the male sex Idania et al. reported for a group of apparently healthy men (*n* = 18) with a mean age of 52 years, like our control group of men, a mean EGF value of 402.7 g/ml, with a standard deviation of 116.88 pg/ml,^36^ also showing no weight effect between these groups (g = 0.0232).

If we compare these data with those reported by Joh Takaki et al. for the Japanese population, with serum EGF concentrations in normal men and women of 780 and 604 pg/ml, respectively,^37^ and more specifically of 417 pg/ml and 572 pg/ml for subjects aged around 60 and 50 years respectively, we find that no differences are observed with our healthy controls and the trend towards higher EGF levels in the context of COVID‐19 is replicated.

In addition, we explored by iconography analysis of correlations, for the study data, where the negative correlation observed between EGF with respect to age (r = −0.3749, *p* = .1030) was not observed in the control group (r = o.0506, *p* = .4135); particularly in the first instance an inverse correlation was observed between EGF with respect to age and EGF with respect to lymphocytes, with a positive correlation between lymphocytes with respect to age, all notable (r > 0.30), but the first order partial correlates indicate an absence of a tripartite correlation (rEGF‐Lymph.Age = −0.2542, *p* = .1776; rEGF‐Age.Lymph = −0.2933, *p* = .1489). This allows us to assume that the observed differences may not be related to age.

It was observed that in COVID patients the serum EGF value correlated positively with the value of the neutrophil‐lymphocyte index, a prognostic indicator of severity and inflammation that has been widely studied in COVID‐19.^38^, ^39^ Likewise, no interference with other clinical and/or analytical data such as age, heart rate, respiratory rate, creatinine, LDH or liver enzymes was observed. To our knowledge, this is the first report to study serum EGF as a biomarker associated with inflammation in patients with severe COVID‐19.

Elucidating the effects of EGF beyond its receptor is still an open question. The well‐documented pro‐inflammatory effect of EGFR,^4^, ^40^, ^41^ in contrast to the anti‐inflammatory effect of EGF observed in LPS‐challenged fibroblasts, even in a hyperglycaemic environment,^42^ exposes a complexity beyond what has been agreed regarding the traditional role of EGF as an inducer of EGFR hetero‐dimerization.

The revaluation of the indices derived from a blood count has made it possible to enhance the diagnostic value of indices such as NLR and PLR, which have been associated with other conditions of a marked inflammatory nature.^43^, ^44^ In the particular case of COVID‐19, they have shown not only value in diagnosis, but also in its stratification and prognosis.^45^, ^46^ These indices are not only valuable in themselves for the information they provide, but also for the optimization and reduction of costs associated with the monitoring of these conditions, with potential effects on public health systems, derived from their generalization.

The results observed in our study, in addition to contributing clinical information to outline the plausible role of EGF in inflammation in the context of COVID‐19; They would contribute to the revaluation of their determinations in other contexts, starting point of the dependence observed in relation to the NLR and PLR, providing from another pathophysiological perspective, a better understanding of the molecular bases of the medical clinic of these conditions.

Our research has two major limitations, the first being that our research is a small sample investigation, and the correlation between EGF and markers of inflammation in COVID‐19 has yet to be explored in more detail; we also lack longitudinal follow‐up results for patients with COVID‐19. However, our study reported the correlation between EGF and markers of inflammation in COVID‐19 for the first time, providing a new perspective extendable to inflammatory lung diseases regarding possible strategies for prevention and treatment of their severe forms.

## 5 CONCLUSIONS

We did not observe a correlation in this pathological scenario with platelet values, which reinforces the hypothesis of the contribution of other tissues to the total serum EGF balance, beyond the aforementioned dependence on platelet lysis. Overall, our study indicates that serum EGF values correlate positively with prognostic inflammatory markers of COVID‐19 severity, and could presumably act as an independent risk factor for the development of inflammation in response to SARS‐CoV‐2.

## AUTHOR CONTRIBUTIONS

Héctor José Pérez Hernández: Conceptualization, Data Curation, Formal Analysis, Research, Methodology, Visualization and Writing (original and final draft). Tania Crombet Ramos: Formal Analysis, Research, Methodology, Supervision, Validation, Visualization and Writing (review and editing).

## CONFLICT OF INTEREST STATEMENT

The authors declare that they and the institutions to which they belong have not received payment from third parties for any aspect of the submitted work; despite this, it points out the existence of scientific relations with the Center for Molecular Immunology.

## Supporting information

Supporting information.
