# Supplementary material for: Notable correlation between serum epidermal growth factor values and inflammatory status in patients with COVID‐19
Source: Immun Inflamm Dis. 2024 Aug 7;12(8):e1355. doi: 10.1002/iid3.1355 (PMC11304898; doi:10.1002/iid3.1355)
Supplement: Supplementary file 1 — Supporting information. [file IID3-12-e1355-s001.docx]

STROBE Statement-Checklist of items to be included in the ***cross-sectional*** survey reports

|  | Article no. | Recommendation | Page  No |
| --- | --- | --- | --- |
| **Title and summary** | 1 | (*a*) Indicate the study design with a commonly used term in the title or abstract. | 1 |
|  |  | (*b*) Provide in the summary an informative and balanced synthesis of what has been done and what has been found. | 2 |
| Introduction | | | |
| Background/ justification | 2 | Explain the scientific background and justification for the research being reported. | 4 |
| Objectives | 3 | Indicate the specific objectives, including pre-established hypotheses. | 5 |
| Methods | | | |
| Study design | 4 | Introduce the key elements of the study design at the beginning of the document. | 5 |
| Configuration | 5 | Describe the relevant setting, locations and dates, including recruitment, exposure, monitoring and data collection periods. | 5 |
| Participants | 6 | (*a*) Indicate the eligibility criteria and the sources and methods of selection of participants. | 5 |
| Variables | 7 | Clearly define all outcomes, exposures, predictors, potential confounders and effect modifiers. Indicate diagnostic criteria, if applicable. | *6* |
| Data sources/ measurement | 8 * | For each variable of interest, indicate the data sources and details of the assessment (measurement) methods. Describe the comparability of assessment methods if there is more than one group. | *6* |
| Bias | 9 | Describe efforts to address potential sources of bias. | 7 |
| Size of the study | 10 | Explain how the size of the study was arrived at | 5 |
| Quantitative variables | 11 | Explain how quantitative variables were treated in the analyses. If applicable, describe which groupings were chosen and why. | 6-7 |
| Statistical methods | 12 | (*a*) Describe all statistical methods, including those used to control for confounding factors. | 6-7 |
|  |  | *(b*) Describe the methods used to examine subgroups and interactions. | 6-7 |
|  |  | (*c*) Explain how missing data were treated | 7 |
|  |  | *(d*) If applicable, describe the analytical methods taking into account the sampling strategy. | 6-7 |
|  |  | (*e*) Describe any sensitivity analysis | - |
| Results | | | |
| Participants | 13 * | (a) Report the number of individuals in each phase of the study, i.e. number of individuals potentially eligible, screened for eligibility, confirmed eligible, enrolled in the study, completing follow-up and analysed. | 8 |
|  |  | (b) Explain the reasons for non-participation in each phase. | n/a |
|  |  | (c) Consider the use of a flowchart | n/a |
| Descriptive data | 14 * | (a) Indicate the characteristics of study participants (e.g. demographic, clinical, social) and information on exposures and potential confounders. | 8 |
|  |  | (b) Indicate the number of participants with missing data for each variable of interest. | n/a |
| Performance data | 15 * | Report the number of summary results or measures | 8 |
| Main results | 16 | (*a*) Provide unadjusted estimates and, if appropriate, estimates adjusted for confounders and their precision (e.g. 95% confidence interval). Clarify which confounders were adjusted for and why they were included. | n/a |
|  |  | (*b*) Report category boundaries where continuous variables have been categorised. | 10 |
|  |  | *(c*) If appropriate, consider translating estimates of relative risk into absolute risk for a meaningful time period. | n/a |
| Other analyses | 17 | Report on other analyses performed (e.g. subgroup and interaction analyses and sensitivity analyses). | 9-12 |
| Debate | | | |
| Main results | 18 | Summary of the main results in relation to the objectives of the study | 14-16 |
| Limitations | 19 | Discuss the limitations of the study, taking into account sources of potential bias or imprecision. Discuss both the direction and magnitude of any potential bias. | 16 |
| Interpretation | 20 | Provide a conservative overall interpretation of the results taking into account the objectives, limitations, multiplicity of analyses, results of similar studies and other relevant evidence. | 14-16 |
| Generalisability | 21 | Discuss the generalisability (external validity) of the study results. | 16 |
| Additional information | | | |
| Funding | 22 | Indicate the source of funding and the role of the funders of this study and, if applicable, of the original study on which this article is based. | 18 |

*Report separately for exposed and unexposed groups.

**Note:** An Explanation and elaboration article discusses each element of the checklist and provides methodological background and published examples of transparent reporting. The STROBE checklist is best used in conjunction with this article (freely available on the PLoS Medicine website at http://www.plosmedicine.org/, Annals of Internal Medicine at http://www.annals.org/ and Epidemiology at http://www.epidem.com/). Information on the STROBE initiative is available at www.strobe-statement.org.
